# Supplementary material for: Causal Relationship between Aging and Anorexia Nervosa: A White-Matter-Microstructure-Mediated Mendelian Randomization Analysis
Source: Biomedicines. 2024 Aug 16;12(8):1874. doi: 10.3390/biomedicines12081874 (PMC11351342; doi:10.3390/biomedicines12081874)
Supplement: Supplementary file 1 [file biomedicines-12-01874-s001.zip › Supplementary Figure/Figure S4/Singe SNP plot of MR analysis for TL to ubm-b-1962.pdf]

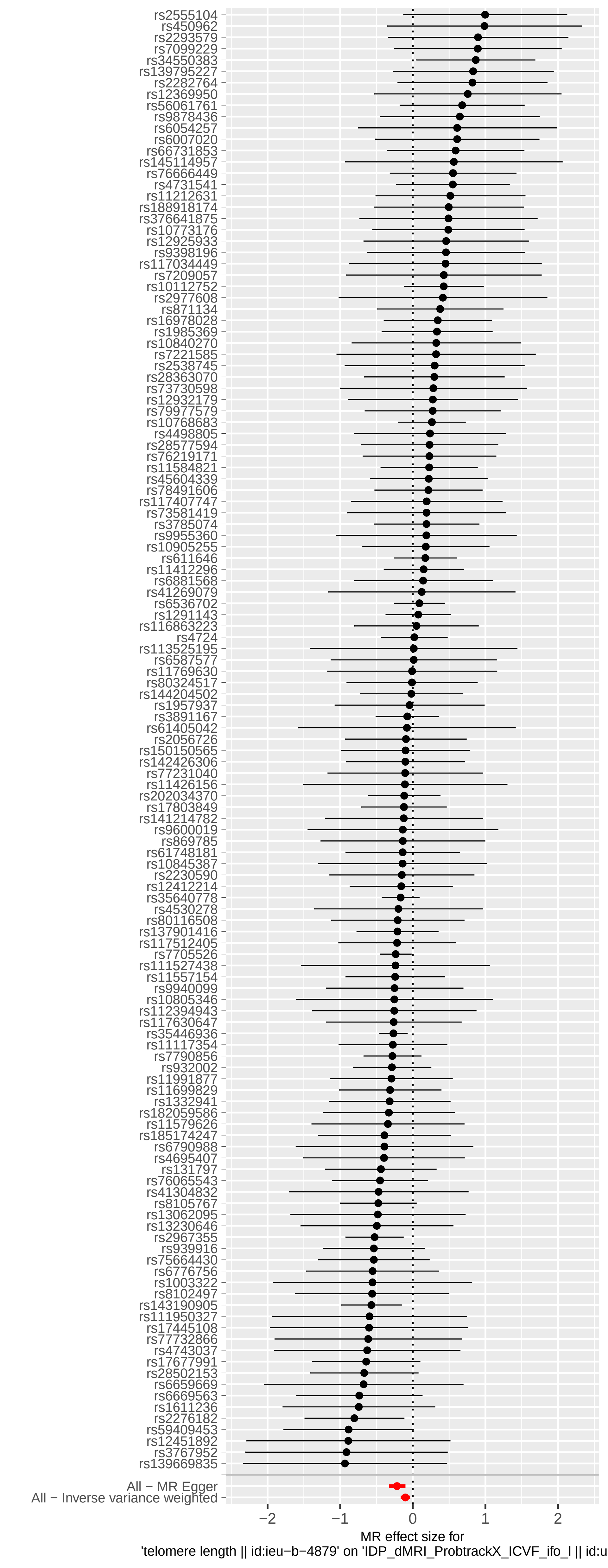
rs2555104
rs450962
rs2293579
rs7099229
rs34550383
rs139795227
rs2282764
rs12369950
rs56061761
rs9878436
rs6054257
rs6007020
rs66731853
rs145114957
rs76666449
rs4731541
rs11212631
rs188918174
rs376641875
rs10773176
rs12925933
rs9398196
rs117034449
rs7209057
rs10112752
rs2977608
rs871134
rs16978028
rs1985369
rs10840270
rs7221585
rs2538745
rs28363070
rs73730598
rs12932179
rs79977579
rs10768683
rs4498805
rs28577594
rs76219171
rs11584821
rs45604339
rs78491606
rs117407747
rs73581419
rs3785074
rs9955360
rs10905255
rs611646
rs11412296
rs6881568
rs41269079
rs6536702
rs1291143
rs116863223
rs4724
rs113525195
rs6587577
rs11769630
rs80324517
rs144204502
rs1957937
rs3891167
rs61405042
rs2056726
rs150150565
rs142426306
rs77231040
rs11426156
rs202034370
rs17803849
rs141214782
rs9600019
rs869785
rs61748181
rs10845387
rs2230590
rs12412214
rs35640778
rs4530278
rs80116508
rs137901416
rs117512405
rs7705526
rs111527438
rs11557154
rs9940099
rs10805346
rs112394943
rs117630647
rs35446936
rs11117354
rs7790856
rs932002
rs11991877
rs11699829
rs1332941
rs182059586
rs11579626
rs185174247
rs6790988
rs4695407
rs131797
rs76065543
rs41304832
rs8105767
rs13062095
rs13230646
rs2967355
rs939916
rs75664430
rs6776756
rs1003322
rs8102497
rs143190905
rs111950327
rs17445108
rs77732866
rs4743037
rs17677991
rs28502153
rs6659669
rs6669563
rs1611236
rs2276182
rs59409453
rs12451892
rs3767952
rs139669835
All − MR Egger
All − Inverse variance weighted
−2
−1
0
1
2
MR effect size for
'telomere length || id:ieu−b−4879' on 'IDP_dMRI_ProbtrackX_ICVF_ifo_l || id:u
